# Transcutaneous auricular neurostimulation to reduce heavy menstrual bleeding in women with and without von Willebrand disease

**DOI:** 10.3389/fmed.2025.1664433

**Published:** 2025-10-08

**Authors:** Christopher J. Czura, Angela C. Weyand, Maureen K. Baldwin, Michael Recht, Melanie A. McWade, Alejandro Covalin, Navid Khodaparast

**Affiliations:** ^1^Spark Biomedical, Inc., Dallas, TX, United States; ^2^Department of Pediatrics, University of Michigan Medical School, Ann Arbor, MI, United States; ^3^Department of Obstetrics and Gynecology, Oregon Health and Science University, Portland, OR, United States; ^4^Yale Center for Bleeding and Clotting Disorders, Yale School of Medicine, New Haven, CT, United States; ^5^National Bleeding Disorders Foundation, New York, NY, United States

**Keywords:** heavy menstrual bleeding, dysmenorrhea, neuromodulation, vagus nerve, pictorial blood assessment chart, menstrual symptoms, clinical trial

## Abstract

Prior studies have revealed that electrical stimulation of the vagus nerve modifies platelet phenotype and reduces blood loss in preclinical models of soft tissue injury. This pilot trial (NCT06064851) sought to determine whether the use of a wearable transcutaneous auricular neurostimulation (tAN) device targeting both the vagus and trigeminal nerve branches correlated with reduced menstrual blood loss in participants with or without von Willebrand disease (VWD). Participants with qualified heavy menstrual bleeding (HMB) gave informed consent to participate in an IRB-approved, decentralized, open-label pilot trial. Participants were followed for two consecutive menstrual cycles. During the baseline menstruation, participants estimated daily blood loss using a validated pictorial blood loss assessment chart (PBAC). During the treatment menstruation, participants self-administered two daily 1-h sessions of tAN daily throughout menstruation and estimated daily blood loss with the PBAC. The PBAC was also used to calculate duration of each menstruation. Student’s paired *T*-test was used to compare mean PBAC scores between menstruations. In participants (*n* = 8) with von Willebrand disease and HMB, use of tAN is associated with 57% lower PBAC scores. Participants with heavy menstrual bleeding of unknown cause (HMBu; *n* = 8) experienced 54% lower PBAC scores while using tAN. Use of tAN also reduced duration of menstruation in both cohorts by 19%. Reductions in menstrual symptoms including cramp and other pain and fatigue and increases in health-related quality of life scores were also noted with use of tAN. tAN activates the vagal and trigeminal networks which are thought to modulate platelet phenotype and lead to improved hemostasis. These pilot results suggest that tAN may be effective in reducing menstrual blood loss in HMB, including those with VWD using concomitant hormonal therapies.

## 1 Introduction

In the United States, 16 million adolescents and adults struggle with heavy menstrual bleeding (HMB). HMB is defined as blood loss that requires changing pads/tampons every 2 h or less, requiring more than one pad/tampon at a time, frequent passing of clots or flooding events, or periods lasting 8 days or more (1). Generally, HMB is determined by personal perception of menstrual blood loss and the related effects on quality of life. Heavy menstrual bleeding adversely affects mood, energy, school/work productivity, social interactions, family life, and sexual health (2–6). HMB contributes to negative workplace productivity; 45.2% reporting absenteeism, 48.4% lacking manager support, and 94.6% lacking specific support or wellness programs for menstrual cycle issues (4, 7). In a recent study, 80.7% (*n* = 26,438) of the respondents reported presenteeism and decreased productivity of an average 23.2 days per year due to menstrual related symptoms (4). HMB may be caused by a variety of factors such as hormonal imbalance, uterine conditions (e.g., fibroids, endometriosis), underlying bleeding disorders including von Willebrand disease (VWD), or other health conditions. Current treatments depend on the underlying etiology, treatment preferences, and pregnancy intentions. In patients with bleeding disorders including von Willebrand disease, HMB is frequently inadequately treated and concomitant therapies are frequently utilized (3). Poor tolerability or acceptability of current treatments suggests that additional non-invasive and non-pharmaceutical solutions are desired (8).

Pre-clinical studies over the past decade have identified a vagal efferent signal to the spleen, termed the “neural tourniquet,” that promotes platelet-mediated hemostasis. Direct cervical vagus nerve stimulation (VNS) reduced bleeding times and shed blood volumes by 45–75% in animal models of soft tissue injury and coagulation factor VIII deficiency (9, 10). Implantable cervical VNS is an FDA-approved treatment for refractory epilepsy, treatment-resistant depression, and chronic ischemic stroke (11). Transcutaneous auricular vagus nerve stimulation (taVNS) has emerged as a non-invasive alternative to invasive cervical VNS and has been applied for headache, migraine, heart failure, gastric motility disorders, asthma, tinnitus, and other conditions (12, 13). taVNS non-invasively targets the auricular branch of the vagus nerve (ABVN), which has projections to various brainstem nuclei (e.g., the nucleus tractus solitarius). Through centrally mediated activation, the ABVN can modulate vagal afferent and efferent networks (13–19). Stimulation of the ABVN releases central nervous system endorphins (20), shifts circulating monocytes to an anti-inflammatory phenotype (21), inhibits pro-inflammatory cytokine release (22), and has sustained antinociceptive effects (23, 24). A recent systematic review and meta-analysis concluded that taVNS is safe and feasible across several indications (25). Transcutaneous auricular neurostimulation (tAN) is similar to taVNS but delivers stimulation to both the ABVN and the auriculotemporal nerve (ATN), a branch of the trigeminal nerve. By targeting the vagus and trigeminal nerves through auricular stimulation, tAN provides a comprehensive, non-pharmacological and non-invasive solution for managing a wide range of menstrual symptoms, from heavy bleeding and cramping pain to mood changes, gastrointestinal discomfort, and associated autonomic disruptions (13, 26–28). The vagus nerve, whose name derives from the Latin word for “wanderer,” provides extensive connectivity between the brain and various organs (29). This crucial pathway carries predominantly afferent signals, with approximately 80% of vagal fibers transmitting information from the body to the brain, while 20% carry regulatory signals in the opposite direction (25). The trigeminal nerve system, with its three major branches (ophthalmic, maxillary, and mandibular), serves crucial sensory and motor functions in the face and head. Beyond these traditional roles, research has revealed its significant involvement in autonomic regulation (13). The trigeminal nerve has extensive connections with the autonomic nervous system and shares important integration centers with the vagus nerve, particularly in the brainstem (30). This trigeminal-vagal interaction creates a powerful therapeutic opportunity, as stimulation of these pathways can synergistically modulate autonomic function (31, 32). The convergence of both vagal and trigeminal branches near the ear surface provides an ideal access point for therapeutic intervention through auricular neurostimulation.

A recent healthy human subject study (NCT05977946) (33, 34) provided first-in-human results for the use of tAN to activate the neural tourniquet pathway. tAN increased ADP-mediated α granule release, expression of activated glycoprotein (GP)IIb/IIIa on the platelet surface and increased maximum clot strength as measured via thromboelastography (TEG), with no reported thrombotic complications (33, 34). Given these first-in-human observations, we sought to assess the therapeutic potential of tAN therapy to decrease menstrual blood loss and other menstrual symptoms in a prospective, decentralized pilot study of people with HMB with and without VWD.

## 2 Methods

### 2.1 Participants

This study was designed as an open label, two-arm, decentralized clinical trial. The research was approved by a central institutional review board (IRB; WCG, Princeton, NJ, United States). The study was registered with ClinicalTrials.gov, identifier number NCT06064851, and was conducted between October 2023 and April 2025. The work was funded by Pathway to Cures, the National Bleeding Disorders Foundation’s venture philanthropy fund. Study devices were provided by Spark Biomedical, Inc., Dallas TX, United States.

**Figure 1:**
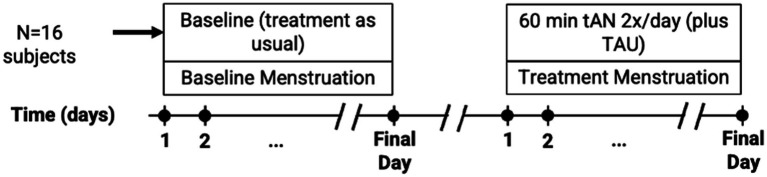
Clinical study design diagram. A total of 16 participants, 8 in each cohort, were recruited who provided data for analysis. Dots along timeline indicate collection of patient-reported outcomes: PBAC was recorded daily, while CMSS and RAND-36 were collected on the final day of each menstrual episode. TAU, treatment as usual.

The primary objective of this study was to determine whether the use of transcutaneous auricular neurostimulation (tAN), when used during menstruation, is associated with reduced menstrual blood loss; secondary objectives were to determine whether use of tAN is associated with improved menstrual symptoms. All subjects were followed for two menstruations. Baseline data were collected during the first menstruation, during which tAN was not administered. tAN was used during the subsequent menstruation, and comparisons were made between baseline and treatment menstruations (Figure 1).

Participants with heavy menstrual bleeding of unknown cause (uHMB; *n* = 8), or heavy menstrual bleeding secondary to von Willebrand disease (VWD + HMB; *n* = 8), were enrolled in this study. Major inclusion criteria included regularly menstruating participants between 18 and 45 years of age, history of menorrhagia as assessed by the Menorrhagia Screening Tool (35), self-reported diagnosis of von Willebrand disease (VWD + HMB cohort), and use of hormone therapy (VWD + HMB cohort) or no use of hormone therapy (HMBu cohort). Participants were asked to not change current hormone therapy, ancillary medications, and supplements in the past 3 months; express a willingness to continue use for duration of study and not start any new medications or homeopathic remedies. Major exclusion criteria included pregnancy within 3 months of enrollment, use of copper intrauterine device, known structural cause of heavy menstrual bleeding, or use of menstrual cups as a method of menstrual blood collection. The etonogestrel implant (Nexplanon, Merck Inc.) was excluded from the VWD + HMB cohort because of prior reports of frequent breakthrough bleeding compared with other hormonal treatments (36). Participants were considered enrolled after signing an informed consent form and completing the screening information electronic case report forms to assess eligibility against the full inclusion/exclusion criteria (Appendix 1 in Supplementary material). Participants who were determined to be ineligible were considered screen failures.

Participants were recruited online from across the US through IRB-approved social media advertisements, which led to a study landing page and a link to the study screener. Participants were not required to attend in-person visits. All data were collected via electronic patient reported outcome forms and electronic case report forms. Study staff contacted preliminarily eligible participants and scheduled a videoconference meeting to review the informed consent form. The Research Coordinator provided instruction on data collection to all participants that met inclusion/exclusion criteria. All study communication with study participants were conducted via the Sponsor’s HIPPA-compliant Zoom platform.

### 2.2 Outcome measures

Pictorial Bleeding Assessment Chart scores were collected daily, and the Cox Menstrual Symptom Scale and RAND-36 were completed on the final day of menstruation. A device usability instrument was completed on the final day of the treatment menstruation.

#### 2.2.1 Pictorial bleeding assessment chart

The primary outcome measure was the Pictorial Bleeding Assessment Chart (PBAC) score (37–39). The PBAC is a validated semi-quantitative tool for patients with HMB to account for menstrual blood loss. Menstrual blood loss is estimated through a selection of graded images of tampons, pads, clots, and flooding events. The participants can directly record the number of used feminine items and the degree of blood saturation. Participants mark a tally next to the images that best match each of the menstruation products they used that day. Additionally, clots size (i.e., dime or quarter size) and incidences of flooding are recorded under the relevant day. Example: study participant #1 on her 2nd menstruation day used 5 pads (2 heavy saturated and 3 moderately saturated), 3 heavily saturated tampons, passed three dime size blood clots, and experienced no flooding events. A total PBAC score of 100 correlates to 80 mL of blood over the duration of the menstruation, which is the classical definition of heavy menstrual bleeding (38, 40). A secondary outcome measure included the duration of menstruation, which was derived from the daily PBAC scores. Participants with a baseline PBAC >150 were advanced to the treatment cycle.

#### 2.2.2 Cox menstrual symptom scale and RAND-36

Menstrual symptoms were assessed on the final day of menstruation using the Cox Menstrual Symptom Scale (CMSS), which assesses frequency and severity of 18 symptoms associated with dysmenorrhea. The symptoms can loosely be organized into three domains: pain (cramps, abdominal pain, headaches, backaches, leg aches and general aches), gastrointestinal (nausea, vomiting, loss of appetite, and diarrhea), and neurocognitive (dizziness, weakness, flushing and sleeplessness). The symptom of facial blemishes does not fit into these domains. The frequency of each symptom is scored on a scale of 0 (did not occur) to 4 (lasted several days), and severity is scored on a scale of 0 (not noticeable) to 4 (very severely bothersome). Improvements in symptom presentation are represented by decreasing values (41). In addition, a general health-related quality of life assessment was conducted using the RAND 36-Item Short Form Health Survey 1.0 (similar to Short Form 36 Health Survey; SF-36), which collects symptom scores across 36 questions organized into eight domains (physical functioning, role limitations due to physical health or emotional problems, energy/fatigue, emotional well-being, social functioning, pain, and general health). Participants enter scores for each question that are then converted to a scale of 0–100, with 0 representing the lowest score and 100 representing the highest possible score; improvements in symptom presentation are represented by increasing values (42).

#### 2.2.3 Device usability

At the end of the study, participants were asked to rate device satisfaction and usability. Satisfaction with the device was graded on a scale of 0–40, with 0–13 considered low satisfaction (difficulty using the device, discomfort, or lack of symptom improvement; usability and integration into daily life were limited); 14–27 moderate satisfaction (the device was somewhat effective and usable, with occasional difficulties or discomfort; integration into daily routine may have been partial); and 28–40 high satisfaction (reported ease of use, comfort, improved symptoms, and seamless integration of the device into daily activities). Usability was rated on a scale of 0–12 across two domains: using the earpiece (ability to connect and disconnect the earpiece to the Patient Controller; to apply the earpiece; and earpiece comfort); and ambulatory factors (less blood loss than typical; ability to wear the device and perform normal daily activities; and ease of integration in typical routine). In these domains, a score of 0–4 was considered low satisfaction, 5–8 moderate satisfaction, and 9–12 was high satisfaction. Participants scored the Patient Controller on a scale of 0–16 based on their ability to identify and fix errors, to increase and decrease stimulation for both channels, to identify low battery status, and to use the therapy timer to manage stimulation sessions. A score of 0–5 was considered low satisfaction, 6–11 moderate satisfaction, and 12–16 was high satisfaction.

### 2.3 Intervention

**Figure 2:**
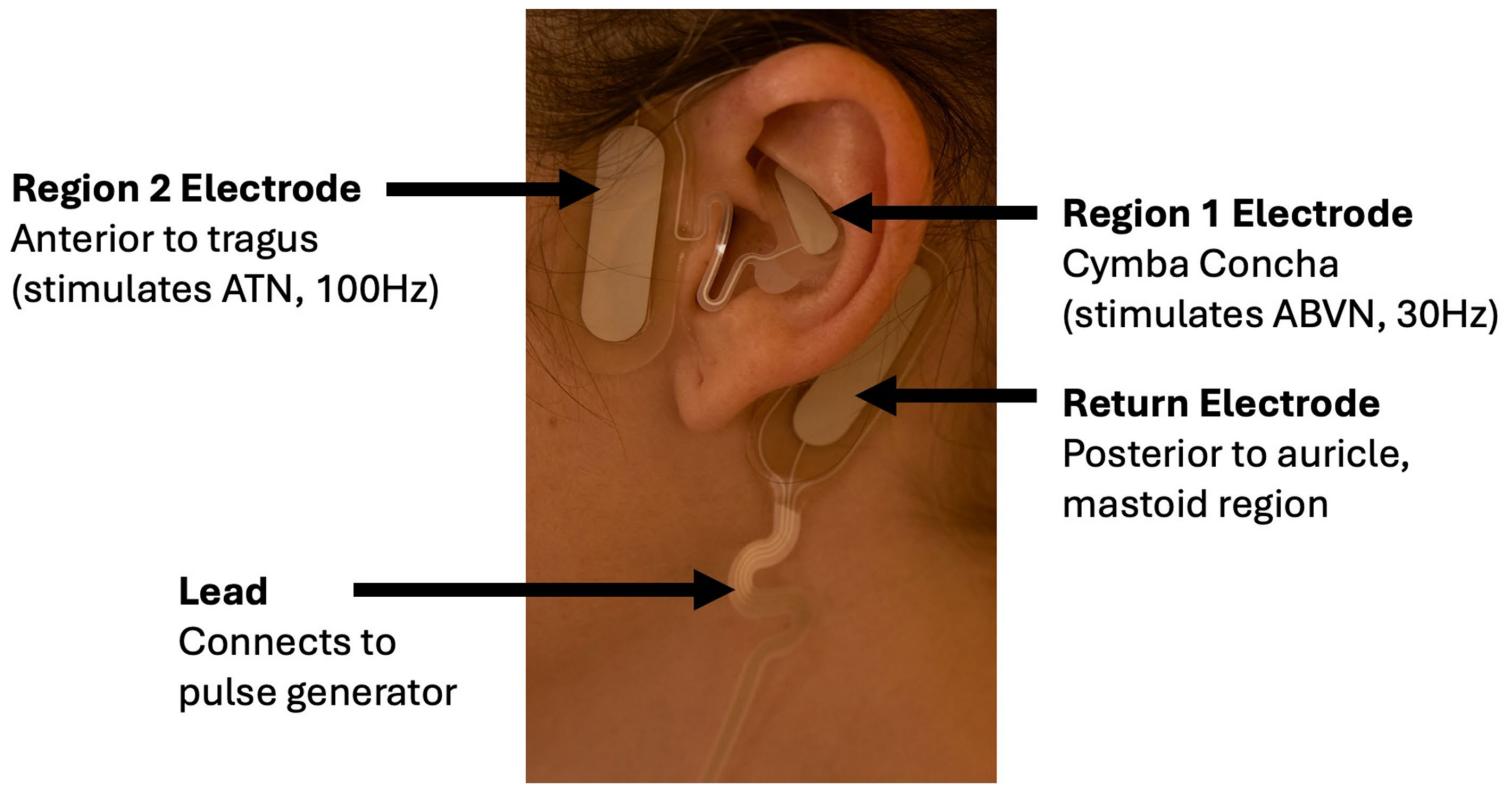
Electrode positioning on the ear of a Volta user. The Region 1 electrode is positioned in the cymba concha to stimulate the ABVN at 30 Hz, while the Region 2 electrode is placed anterior to the tragus to stimulate the ATN at 100 Hz. The return electrode is positioned posterior to the auricle near the mastoid region, and a lead connects the electrode to the external pulse generator.

The Volta is derived from the Sparrow Ascent™, a Class II medical device cleared by the Food and Drug Administration (FDA) to manage the symptoms of opioid withdrawal (K230796) (31) (Figure 2). Two individual stimulation frequencies were used: 30 Hz at the cymba concha to stimulate the auricular branch of the vagus nerve (ABVN) and 100 Hz at the temporal mandibular region to target the auriculotemporal nerve (ATN), a branch of the trigeminal network (32). The pulse duration was set to 250 μs with a duty cycle of 5 min ON followed by 10s OFF. The stimulation intensities (milliamps; mA) were set to 0 mA for both regions. Participants were instructed to increase the intensity in each region until the stimulation was perceivable by activating the appropriate (up or down) button.

### 2.4 Experimental procedures

To determine whether tAN is associated with reduced menstrual blood loss, participants used tAN twice per day, 1 h each, during menstruation. The start of menstruation was defined as the first day during which tampons or pads were needed, and the last day of menstruation was defined as the last day those products were needed. After the final day of the baseline menstruation, participants were shipped a Volta™ device. Participants were trained via videoconference on device use. Participants applied an electrode to the left ear; the first stimulation session was delivered in the morning and the second in the evening. After 1 h for each session, stimulation concluded, and the Patient Controller was turned off and disconnected. The earpiece was removed and discarded.

### 2.5 Statistical analysis

With no prior human data on the use of neuromodulation to improve hemostasis, a formal power calculation could only be performed using data from animal models; with a statistical significance level of *p* < 0.05, 8 subjects provided 90% power for detecting 50% less blood loss with 30% variability. All subjects who received devices and returned data were included in the analysis. Baseline menstruation scores were compared to treatment menstruation scores with the paired Student’s *T* test.

## 3 Results

**Figure 3:**
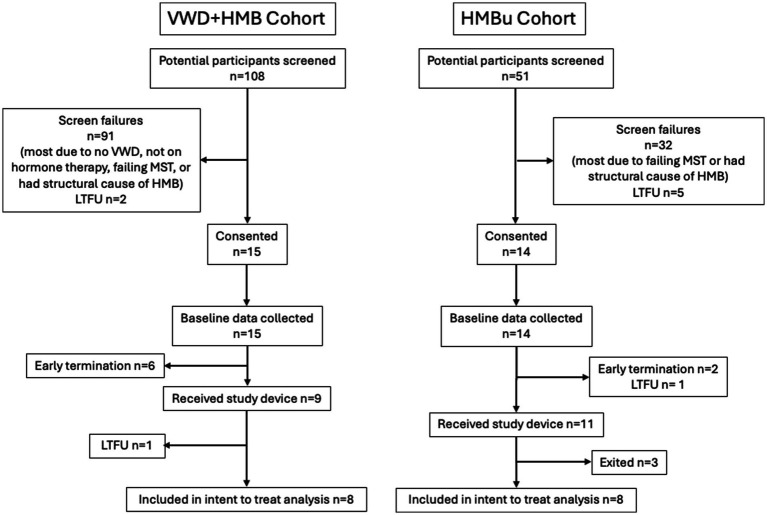
CONSORT flow diagram. Early terminations occurred due to inconsistent data reporting (*n* = 4), PBAC < 150 (*n* = 3), or menstruation 15 days or longer (*n* = 1). Participant exits after receiving study device occurred because they were deemed non-compliant with protocol (*n* = 2) or were unable to position the device properly (*n* = 1). VWD, von Willebrand disease; HMB, heavy menstrual bleeding; UHMB, HMB of unknown cause; MST, menorrhagia screening tool; LTFU, lost to follow-up.

Of the 108 potential subjects screened for the VWD + HMB cohort, *n* = 9 ultimately received the study device; one was lost to follow-up after the treatment cycle without data collection, resulting in *n* = 8 included in the analysis. Of the 51 potential subjects screened for the HMBu cohort, *n* = 11 ultimately received the study device; 2 were exited before analysis due to inconsistent data reporting, and one was unable to reliably apply the electrode; *n* = 8 were included in the analysis (see CONSORT diagram, Figure 3). Demographics of the study participants in each cohort are listed in Table 1.

**Table 1:** Key patient characteristics in both cohorts.

Variables	Categories	VWD cohort	HMBu cohort
Age, years	Mean	37.9 ± 3.8	32.0 ± 9.8
	Range	31–43	18–44
Race	White	3 (37.5%)	2 (25.0%)
	African American	4 (50.0%)	6 (75.0%)
	Multiracial	1 (12.5%)	0 (0.0%)
Ethnicity	Hispanic	2 (25.0%)	2 (25.0%)
	Non-Hispanic	6 (75.0%)	6 (75.0%)
Prior treatments for anemia	Yes	3 (37.5%)	1 (12.5%)
	No	5 (62.5%)	7 (87.5%)
Typical duration of menstruation, days		7 ± 1.6	6.9 ± 0.7
Hormone therapy	Oral EE/P	5 (62.5%)	0 (0.0%)
	LNG-IUD	3 (37.5%)	0 (0.0%)
	None	0 (0.0%)	8 (100.0%)
Height (cm)		170.9 ± 11.4	166.1 ± 5.6
Weight (kg)		95.5 ± 24.3	70.8 ± 9.5

Data are mean ± standard deviation or *n* (%). Multiracial participants identified as White and Black. Oral EE/P, oral ethinyl estradiol/progestin; LNG-IUD, levonorgestrel intrauterine device.

### 3.1 Changes in menstruation

**Figure 4:**
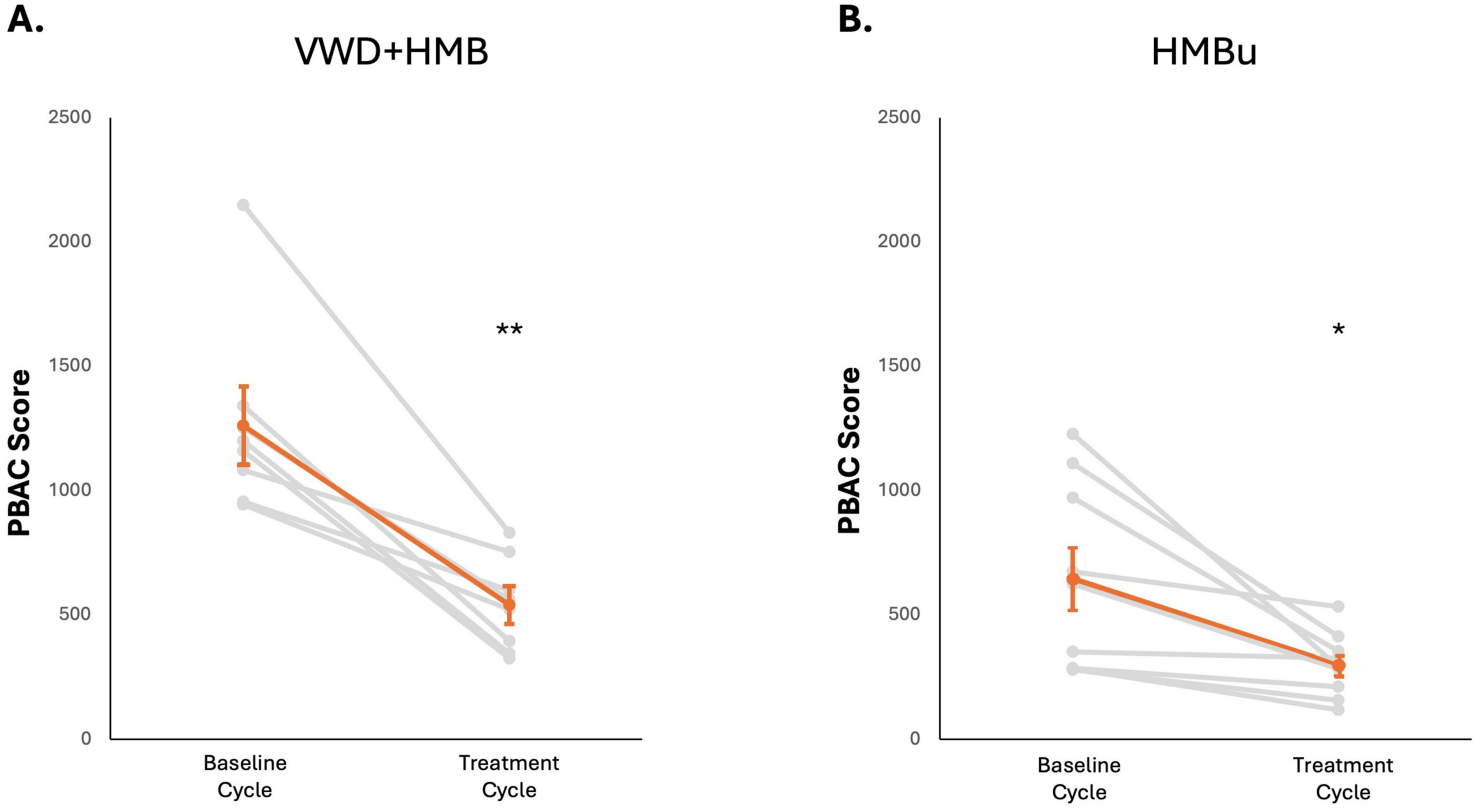
Effects of tAN on menstruation. In all panels, responses of individual participants are shown in grey, mean and standard error are shown in orange. **(A)** tAN is associated with a 57.1% reduction in menstrual blood loss in participants with von Willebrand disease and HMB (*n* = 8; **p < 0.01). **(B)** tAN is associated with a 53.8% reduction in menstrual blood loss in participants with HMB of unknown cause (*n* = 8; *p < 0.05).

**Figure 5:**
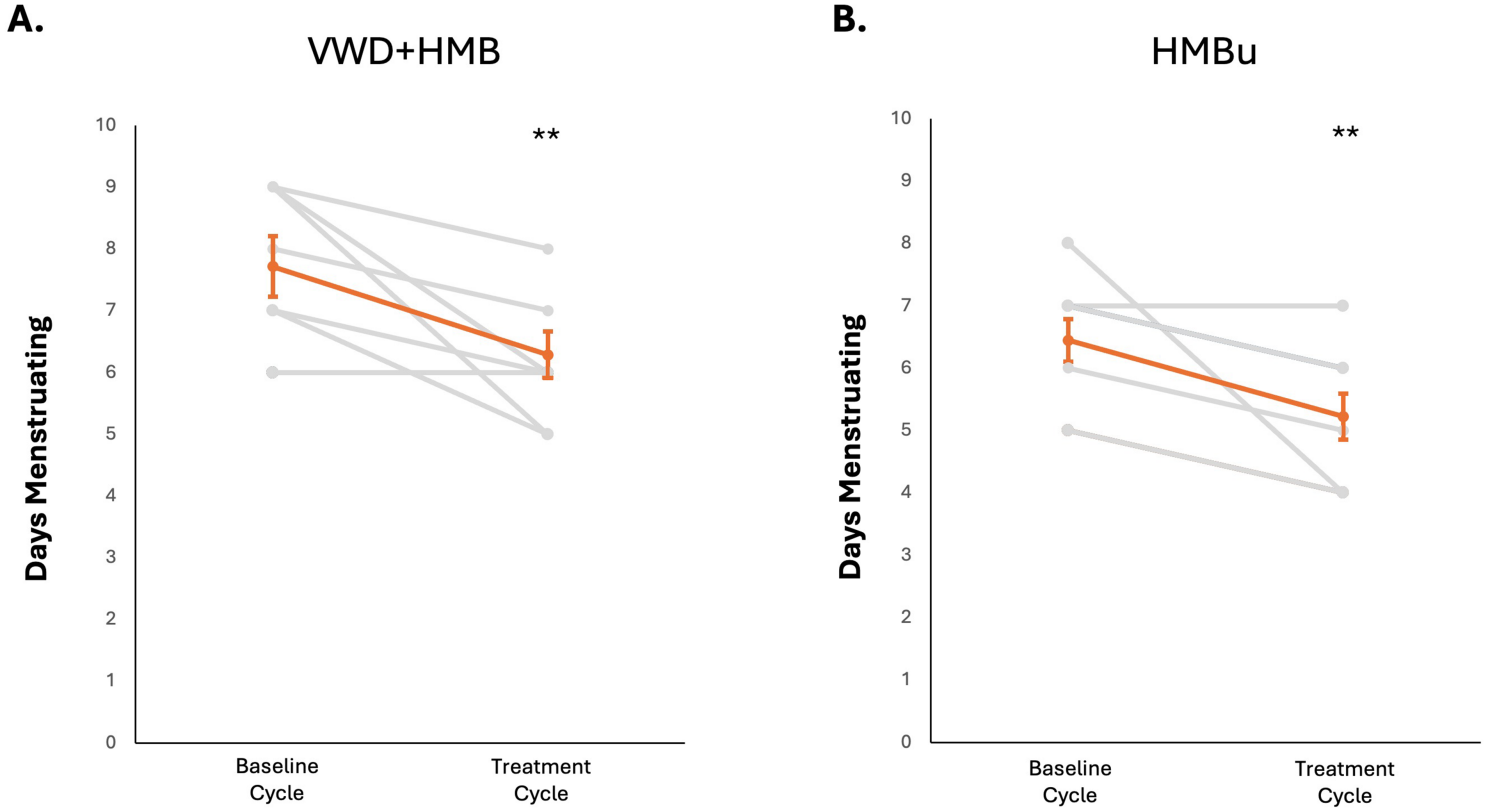
Effects of tAN on duration of menstruation. In all panels, responses of individual participants are shown in grey, mean and standard error are shown in orange. **(A)** tAN is associated with a 19.7% reduction in duration of menstruation in HMB+VWD participants (*n* = 8; **p < 0.01). **(B)** tAN is associated with a 19.6% reduction in duration of menstruation in HMBu participants (*n* = 8; **p < 0.01).

To determine whether use of tAN was associated with reduced menstrual blood loss, participants used the PBAC to estimate menstrual blood loss in a baseline menstruation (no stimulation), and again in a second menstruation during which tAN was delivered. The VWD + HMB cohort (*n* = 8) had a mean baseline menstrual blood loss PBAC score of 1,260 ± 136; the mean PBAC score while using tAN was 541 ± 65 (*p* < 0.05 vs. baseline), 57.1% lower than baseline (Figure 4A). The HMBu cohort (*n* = 8) had a mean menstrual blood loss PBAC score of 647 ± 135; the mean PBAC score while using tAN was 299 ± 46 (*p* < 0.01 vs. baseline), 53.8% lower than baseline (Figure 4B). Use of tAN reduced duration of menstruation in VWD + HMB participants by 19.7% (7.6 ± 0.5 days to 6.1 ± 0.4 days, *n* = 8; *p* < 0.01; Figure 5A), and in HMBu participants by 19.6% (6.4 ± 0.4 days to 5.1 ± 0.4 days, *n* = 8; *p* < 0.01; Figure 5B). Participants in both cohorts used statistically significantly fewer menstrual products, and VWD + HMB participants passed statistically significantly fewer clots, while using tAN (Table 2).

**Table 2:** Menstrual symptoms reported via the Cox Menstrual Symptom Scale, and health-related quality of life endpoints as reported by the RAND-36.

Outcome Measure	Variables	VWD + HMB			HMBu		
		Baseline	w/tAN	*p*-value	Baseline	w/tAN	*p*-value
Product use	Pads + tampons	70 ± 17	41 ± 4	**0.0001**	38 ± 3	28 ± 2	**0.006**
Clots		25 ± 8	13 ±3	**0.027**	12 ± 5	7 ± 2	0.096
CMSS	Overall	43 ± 7	25 ± 6	**0.035**	55 ± 13	36 ± 11	**0.023**
	Frequency	23 ± 4	13 ± 4	**0.016**	29 ± 6	19 ± 5	**0.015**
	Severity	20 ± 4	12 ± 3	0.063	26 ± 6	38 ± 6	**0.017**
	Pain frequency	12 ± 1	6 ± 1	**0.024**	13 ± 2	9 ± 2	**0.012**
	Pain severity	11 ± 1	5 ± 1	**0.024**	13 ± 2	8 ± 2	**0.005**
	GI frequency	3 ± 1	1 ± 1	0.102	4 ± 1	2 ± 1	0.058
	GI severity	3 ± 1	1 ± 1	0.080	3 ± 1	2 ± 1	0.051
	Neurocog frequency	6 ± 1	3 ± 1	0.056	9 ± 3	7 ± 2	0.075
	Neurocog severity	6 ± 1	3 ± 1	0.123	9 ± 3	7 ± 3	0.108
RAND-36	PF	86 ± 5	88 ± 5	0.366	73 ± 12	83 ± 5	0.201
	PH	69 ± 11	97 ± 3	**0.013**	66 ± 16	75 ± 13	0.299
	EP	58 ± 12	96 ± 4	**0.013**	63 ± 17	83 ± 12	0.203
	EF	39 ± 6	54 ± 7	**0.050**	41 ± 8	45 ± 7	0.299
	EWB	73 ± 3	73 ± 5	0.500	62 ± 7	62 ± 8	0.474
	SF	77 ± 9	88 ± 7	**0.032**	73 ± 8	67 ± 9	0.204
	Pain	71 ± 6	83 ± 3	0.075	63 ± 9	59 ± 8	0.294
	GH	66 ± 5	71 ± 4	0.142	68 ± 8	66 ± 7	0.284

Data are presented as mean ± SEM; *p* < 0.05 highlighted in bold font. CMSS, Cox menstrual symptom scale; GI, gastrointestinal; Neurocog, neurocognitive; PF, physical functioning; PH, role limitations due to physical health; EP, role limitations due to emotional problems; EF, energy and fatigue; EWB, emotional well-being; Pain, general body aches and pains; GH, general health.

### 3.2 Changes in menstrual symptoms

The CMSS was used to determine whether the use of tAN during menstruation was associated with decreased menstrual symptoms. VWD + HMB participants experienced an overall reduction in CMSS scores of 41.6% (baseline = 43 ± 7 vs. with tAN = 25 ± 6; *p* < 0.05) and a 41.9% reduction in symptom severity score (baseline = 20 ± 4 vs. tAN = 12 ± 3; *p* < 0.05). The HMBu participants experienced an overall reduction in CMSS scores of 33.7% (baseline = 55 ± 13 vs. with tAN = 36 ± 11; *p* < 0.01), a 33.3% reduction of symptom severity scores (baseline = 26 ± 6 vs. tAN = 18 ± 6; *p* < 0.05), and a 34.1% reduction of symptom frequency scores (baseline = 29 ± 6 vs. tAN = 19 ± 5; *p* < 0.05). CMSS domains assessing pain, gastrointestinal discomfort, and neurocognitive issues were also analyzed; only scores for frequency or severity of pain were statistically significantly reduced in both cohorts (Table 2). Analyses of CMSS scores in all participants revealed significant reductions across all subdomains. Notably, cramp pain was significantly reduced across all study participants.

### 3.3 Changes in health-related quality of life

The RAND-36 was used to determine whether the use of tAN during menstruation was associated with increased health-related quality of life. In the VWD + HMB cohort, RAND-36 scores improved with use of tAN across multiple domains, including role limitations due to physical health, role limitations due to emotional health, energy and fatigue, and social functioning. Analyses of the HMBu cohort showed no statistically significant changes in any RAND-36 domain (Table 2). Notably, VWD + HMB participants reported spending 52.2% less extra time in bed while using tAN (baseline = 12.6 ± 3.3 h vs. tAN = 6.0 + 4.9 h, *p* = 0.01), and HMBu participants reported spending 31.5% less extra time in bed while using tAN (baseline = 10.1 ± 1.8 h vs. tAN = 6.9 ± 1.5 h, *p* = 0.01; data not shown).

### 3.4 Device usability

Device usability is a critical determinant of treatment outcomes, as it directly influences patient adherence, accuracy of use, and long-term engagement with therapy. Intuitive and ergonomically designed devices may reduce user error, support consistent treatment dosing, and enhance overall therapeutic efficacy, ultimately minimizing the risk of noncompliance or discontinuation. The 16 participants who completed the study reported mean+/-SE satisfaction scores of 34 ± 1 on a scale of 0–40, with only one participant assigning a moderate satisfaction score of 24 (data not shown). Usability of the device in terms of ambulatory factors received a mean+/-SE score of 11 ± 0.5 on a scale of 0–12, with only one participant reporting a moderate satisfaction score of 7 (data not shown). Usability of the Patient Controller received a mean+/-SE score of 14 ± 0.5 on a scale of 0–16, with only one participant reporting a moderate satisfaction score of 7 (data not shown). Usability of the earpiece received a mean+/-SE score of 10 ± 0.4 on a scale of 0–12, with three participants reporting a moderate satisfaction score of 8, and one participant reporting a moderate score of 5 (data not shown). Consistent with these results, it is of note that one participant in the HMBu cohort reported repeated difficulty in applying the electrode to a narrow cymba concha region and withdrew from the study prior to their treatment menstruation. Across both cohorts, participants applied a mean of 1.1 ± 0.3 mA on the inner electrode, and 1.5 ± 0.4 mA on the outer electrode.

### 3.5 Adverse events

A total of 4 device-related adverse events (AEs) were recorded in this study across four subjects. Three subjects reported mild skin irritation and sensitivity due to the inner electrode not fitting properly. A fourth subject reported mild irritation and sensitivity around the top of the ear due to abrasion from an extension of the electrode that arches over the ear. Symptoms resolved after discontinued use of the earpiece; no medical intervention was required. One serious AE (SAE) was reported by a subject who reported to a hospital and received a blood transfusion due to anemia. This SAE occurred after baseline menstruation but before tAN was administered and was thus considered unrelated to device use. No data were collected from this participant during the treatment menstruation and thus could not be included in the analysis.

## 4 Discussion

This pilot study demonstrates feasibility and promising therapeutic efficacy greater than 50% blood loss reduction in HMB among those with and without a bleeding disorder, a clinically meaningful result (43). Use of tAN also demonstrated meaningful reduction in total duration of menstruation. Participants reported significant improvements in the frequency and severity of menstrual symptoms, especially pain, as assessed by the CMSS, as well as improved health-related quality of life scores in the RAND-36 for the VWD + HMB cohort. Notably, participants reported spending 30–50% less extra time in bed due to menstrual symptoms. These promising findings are the first in human therapeutic application of tAN to improve menstrual health.

Current treatments depend on the underlying etiology, treatment preferences, and contraception needs. First line medical treatments include hormonal therapies such as oral, transdermal, or vaginal combined estrogen/progestins, progestin intrauterine devices (IUD), injectables, and subdermal implants (44); and non-hormonal therapies, including anti-fibrinolytic drugs (45); or non-steroidal anti-inflammatory drugs (NSAIDs) (46). Hormonal therapies are highly successful and satisfactory but are sometimes associated with adverse effects that include breakthrough bleeding, nausea, weight gain headaches, edema, and depression (47, 48). Non-hormonal treatments are recommended to reduce menstrual blood loss in patients for whom hormonal treatment is not suitable or preferred (44, 49). For example, antifibrinolytics like tranexamic acid (TXA) are generally well-tolerated, but can cause additional discomfort, headache, back pain, nausea, and diarrhea (50). For people with extreme cases of HMB or underlying bleeding disorders, surgical approaches or blood coagulation factor replacement therapy may be warranted. Surgical approaches include endometrial ablation and hysterectomy. Hysterectomy is a major surgery with associated risks. Additional therapeutic options that do not alter the hypothalamic pituitary axis are needed as alternatives to hormonal medications, or as adjunct therapy for those with inadequate treatment outcomes.

Hormone therapy can often result in complete cessation of menstruation for some users. However, menstruation can occur with some formulations, especially oral contraceptives that include a placebo week. In this study, 62.5% of VWD + HMB cohort participants reported taking oral hormonal therapies as per recommended dosage and administration in the package insert. The remainder of the cohort had a hormone-eluting intrauterine device (IUD) placed. Despite these hormone therapies, VWD + HMB participants reported exceptionally high baseline PBAC scores (mean 1260 ± 136), well in excess of the clinical definition of heavy menstrual bleeding (PBAC > 100). Although this may not be representative of all women who have HMB, it represents a scenario in which hormone therapies may be insufficient in reducing menstrual blood loss. PBAC scores were reduced by >50%, a reduction equivalent to that seen with TXA (51). The HMBu cohort in this study, who were not on any form of hormone therapy, had significantly lower baseline PBAC scores than the VWD + HMB cohort (data not shown), which is not unexpected given the bleeding disorder in the latter cohort. Nonetheless, the HMBu cohort experienced a reduction in PBAC score similar in magnitude (>50%) as that observed in the VWD + HMB cohort. These observations suggest that the neural tourniquet pathway in humans is independent of von Willebrand factor, and activation of the pathway can improve hemostasis in the absence or presence of hormone therapy. Participants’ menstruations in both cohorts were ~1.5 days shorter while using tAN as compared with baseline menstruation, an effect not observed with TXA (52).

Participants in both cohorts in this study also experienced significant improvements in menstrual symptoms or health-related quality of life with the use of tAN. In the VWD + HMB cohort, use of tAN significantly improved severity and overall experience of menstrual symptoms on CMSS and improved several domains on the RAND-36. In contrast, use of tAN did not significantly change RAND-36 outcomes in the uHMB cohort, but the CMSS did reveal that use of tAN correlated with improvements in the frequency, severity and overall experience of menstrual symptoms as compared with baseline. A CMSS subdomain analysis revealed that pain is significantly reduced with the use of tAN. The effects of tAN on dysmenorrhea as measured by CMSS are similar to a prior study using transcutaneous auricular vagus nerve stimulation (taVNS) (27). In that study, participants received 30 min/day of taVNS at 1 Hz over 10 consecutive days between menstruations. The current study applied 60 min of tAN at 30 Hz to the ABVN (plus 100 Hz to the ATN) twice per day during menstruation. Wang et al. reported ~50% reduction in two menstruations following the use of taVNS, suggesting a persistence of effect on menstrual symptoms over time; the current study did not follow participants into subsequent menstruations. Moving forward, delivering tAN for consecutive cycles, during the luteal and menstrual phases, could demonstrate sustained or gradual improvements in menstrual symptoms. Thus, future treatment dosing studies are warranted.

Usability of the Volta device was rated favorably overall among participants in this study. Users did encounter some difficulty with the earpiece, in particular the inner electrode that is placed on the cymba concha to target the ABVN. The use of tAN during menstruation was safe over the duration of this study, with adverse events limited to minor skin irritation resulting from electrode placement in four of the participants. These observations are consistent with previous findings (31, 32). Transcutaneous auricular neurostimulation has an improved safety profile compared to implanted vagus nerve stimulators, which have been implanted in over 125,000 worldwide since the 1990s. Unlike implanted VNS, which have acute side effects during the “on” phase of stimulation such as cough, hoarseness, voice alteration, and paresthesia, tAN has minimal side effects due to its non-invasive nature (25). The Volta earpiece design with hydrogel electrodes minimizes skin irritation and discomfort and improves current distribution. Notably, no adverse events related to thrombosis have been reported in studies of cranial nerve stimulation, including implanted VNS devices. Indeed, an implanted VNS system was recently approved by the FDA to reduce upper extremity motor deficits and improve motor function during rehabilitation therapy in chronic ischemic stroke patients, a population that may be at increased risk of thrombotic events (53). The non-invasive nature of tAN overcomes many of the shortcomings of traditional VNS systems, including the expense and risks associated with surgical implantation (54).

The mechanism of tAN modulating hemostasis in humans has not been fully elucidated, however the results align with the prior preclinical studies (9, 10). In murine models of soft tissue injury vagus nerve stimulation (VNS) reduces blood loss; including mice deficient in coagulation factor VIII, a genetic deficiency similar to hemophilia A. VNS shortens the reaction (R) time on thromboelastography (TEG) and increases production of thrombin/anti-thrombin in blood shed from the wound (but not systemically) (9). VNS induces the release of acetylcholine in the spleen, which binds platelet acetylcholine receptors and triggers an influx of calcium. Platelets “primed” with elevated calcium levels are more responsive to pro-coagulant triggers at an “injury site” and have a greater increase of P selectin surface expression as compared with platelets from VNS-naïve mice (10). A recent randomized, sham-controlled, double-blinded study of tAN and taVNS in healthy human subjects suggests that transcutaneous neuromodulation strategies can improve laboratory hemostasis in humans (33, 34). Blood samples collected from subjects in this study revealed decreased R time and increased clot firmness as measured by the maximum amplitude parameter after stimulation as compared with sham. Platelet surface marker expression was measured by flow cytometry after *ex vivo* activation with platelet agonists; following stimulation, platelets responded to thrombin with increased surface expression of P selectin, while adenosine diphosphate (ADP) increased expression of glycoprotein IIb/IIIa compared with sham (33–34). Whether these changes occur in women with HMB, with or without VWD, requires further study.

Preclinical studies that have demonstrated VNS-induced improvements in hemostasis have leveraged devices that apply electrodes directly to the cervical vagus nerve, activating signals to the spleen that release acetylcholine and prime platelets (10). In this study, we used a transcutaneous approach to activate the vagus and trigeminal nerves on and around the ear. Stimulation of the auricular branch of the vagus nerve elicits responses in regions of the nucleus tractus solitarii (NTS) that are associated with cardiovagal outflow (55). The NTS is also associated with the regulation of other peripheral organs, including the spleen and immune system (54, 56). The neuroanatomical connection between cranial nerves around the human ear and platelets is not known; we hypothesize that tAN activates afferent signals to the NTS, which then activates efferent vagus nerve signals to the spleen via the splenic nerve (22, 57, 58).

The current study is not without limitations that should be addressed in future studies. A sample size of 8 per cohort is generally regarded as relatively small as compared with pharmaceutical studies. Subjects were followed for only one baseline menstrual episode, compared with 2–3 episodes in most trials (51), and use of concomitant therapies was heterogenous. Assessment of the underlying etiology of HMB and diagnosis of von Willebrand disease were not confirmed with medical chart reviews or direct testing methods. This was an open label study with baseline measurements taken during one menstruation before using the device and then in following menstruation with the use of tAN. As a pilot investigation, the design allowed for preliminary evaluation of both treatment effect and safety profile of a novel therapy, but the absence of a proper control group limits the ability to distinguish true therapeutic benefit from placebo effect or natural variability. The short duration of the study was an additional limitation, as was lack of bloodwork to directly assess the effects of tAN on hemostasis in these populations. Future studies should include larger sample sizes, blinded and sham-controlled arms, treatment during multiple consecutive menstruations, stratification by bleeding etiology, and incorporation of laboratory-based assessments of coagulation and platelet function.

## 5 Conclusion

This is the first study demonstrating use of a transcutaneous auricular neurostimulator during menstruation is associated with decreased menstrual blood loss among those with and without underlying bleeding disorder, including those using adjunct hormonal therapies. Across all study participants, tAN was safe, well-tolerated, and delivered clinically meaningful reductions in menstrual blood loss, menstrual symptoms, and quality of life. tAN activates the vagal and trigeminal networks which are hypothesized to modulate platelet phenotype and lead to improved hemostasis. This treatment modality offers a potential alternative to medications or surgery for women with HMB. A future FDA pivotal trial will be required to demonstrate safety and efficacy of tAN to treat HMB. Additional studies of tAN as a non-pharmaceutical, non-invasive approach to improve hemostasis in other bleeding disorders, as well as in surgery and trauma, are ongoing.

## Data Availability

The raw data supporting the conclusions of this article will be made available by the authors, without undue reservation.
