# Supplementary material for: Transcutaneous auricular neurostimulation to reduce heavy menstrual bleeding in women with and without von Willebrand disease
Source: Front Med (Lausanne). 2025 Oct 8;12:1664433. doi: 10.3389/fmed.2025.1664433 (PMC12540315; doi:10.3389/fmed.2025.1664433)
Supplement: Supplementary file 1 [file Data_Sheet_1.docx]

Appendix 1

**Inclusion Criteria**

1. Regularly menstruating female participants between 18-45 years of age
2. Typical length of menstruation ranging from 7 to 14 days
3. History of menorrhagia as assessed by the Menorrhagia Screening Tool
4. For participants who have been diagnosed with von Willebrand Disease Type 1: On hormone therapy (with the exception of Nexplanon) for at least three months and willing to continue use for the duration of the study
5. For participants with idiopathic heavy menstrual bleeding: No use of hormone therapy for at least the past three months
6. No changes to all current medications and supplements in the past three months, willingness to continue use for duration of study, and not start any new medications or homeopathic remedies
7. Reliable access to an internet-enabled device to complete required questionnaires
8. Willingness to consistently use the same brand of tampons and/or pads throughout duration of the study

**Exclusion Criteria**

1. Pregnancy within three months of enrollment
2. Lactating at the time of enrollment
3. Antifibrinolytic use within 30 days of enrollment
4. Acquired bleeding disorder
5. Use of anticoagulants (i.e., Aspirin, Warfarin, Coumadin, etc.) including platelet inhibitors for 30 days prior to enrollment
6. Use of the Copper intrauterine device within the past 3 months
7. Known structural cause of heavy menstrual bleeding
8. Use of menstrual cups as a method of menstrual blood collection
9. Participant has a history of chronic tobacco use or has ingested nicotine via smoking, vaping, smokeless tobacco, or nicotine patches in the past three months
10. Participant has received a blood transfusion within 30 days prior to study
11. Participant has a history of epileptic seizures
12. Participant has a history of neurologic diseases or traumatic brain injury
13. Participant has presence of devices (e.g., pacemakers, cochlear prostheses, neurostimulators)
14. Participant has abnormal ear anatomy or ear infection present
15. Participant has any other significant disease or disorder which, in the opinion of the Investigator, may either put the participants are risk because of participation in the trial, or may influence the result of the trial, or the participant’s ability to participate in the trial

Datasets are available on request:
The raw data supporting the conclusions of this article will be made available by the authors, without undue reservation.
